# The cervical cancer related distribution, coinfection and risk of 15 HPV types in Baoan, Shenzhen, in 2017–2023

**DOI:** 10.1186/s12985-024-02439-2

**Published:** 2024-07-29

**Authors:** Rukai Li, Weiwei Meng, Yunhai Zuo, Yanli Xu, Shaonan Wu

**Affiliations:** Shenzhen Baoan Shiyan People’s Hospital, No. 11 Jixiang Road, Shiyan, Baoan District, Shenzhen, 518108 China

**Keywords:** HPV, TCT, Colposcopy, Cervical cancer, Coinfection

## Abstract

Cervical cancer is one of the most common malignant tumours. Human papillomavirus (HPV) infection is the main cause of this cancer so that it could be prevented by screening and early treatment. Developing reginal screen protocols of maximum public health efficacy requires in-depth understandings of local HPV distribution and consequential cancer risks. Therefore, test results of HPV genotyping, cytology testing (TCT) and colposcopy inspection with biopsy were collected in this retrospective research. Data included by this research involved 63,906 women received screen related tests from Shenzhen Baoan Shiyan People’s Hospital and the subsidiary institutes between 2017.01 and 2023.05. 10,238 colposcopies were performed in this period collecting 8,716 samples and 814 high-grade CIN were discovered. Within the 763 high-grade CIN cases with both TCT and HPV testing results, 232 were tested cytologically normal but only 30 were negative in HPV test. Besides, the rates of high-grade CIN observed in coinfection were all lower than the estimated rates generated from related single infection. HPV 52, 58 and 16 were found to be the most common types in Baoan, Shenzhen. The result also suggested that HPV coinfections should not increase risk for cervical cancers.

**Supplementary Information:**

The online version contains supplementary material available at 10.1186/s12985-024-02439-2.

## Introduction

Cervical cancer is the fourth most common female malignant tumour [1, 2] with more than 500,000 new cases worldwide causing about 250,000 death in China each year [3]. Standard screening procedure for cervical cancer includes ThinPrep cytologic tests (TCTs), human papillomavirus (HPV) infection test and colposcopy guided biopsy [4]. HPV infection is one of the most common sexually transmitted diseases and a primary cause of cervical cancer [5]. Based on carcinogenicity, subtypes HPV can be divided into high-risk types, including 16, 18, 31, 33, 35, 39, 45, 51, 52, 56, 58 and 59, and low-risk types such as 6, 11, 66, 73 and 82 [6]. Low-risk HPV types often cause warts not progressing into cancers.

Cervical cancers are preventable through controlling HPV infections. HPV are mainly transmitted through skin-to-skin or skin-to-mucosa contact by targeting mucosal tissues, causing cancers in vulva, vagina, penis or anus [7]. Apart from cervical cancer screening, another effective measure to prevent cervical cancer is HPV vaccination [8]. By the time this article was written, there were 3 HPV vaccines available in the market, covering HPV types 16, 18 (bivalent) and 6, 11, 16, 18 (quadrivalent) and 6, 11, 16, 18, 31, 33, 45, 52, 58 (nonavalent) respectively. However, these vaccines were not included by national immunisation program due to high price and insufficient supply [9]. Moreover, HPV prevalence could vary significantly in different regions [10] and types covered by the vaccines might not align to the local type distribution profile. An in-depth knowledge of local HPV characteristics would be crucial for establishing region specific cervical cancer preventing protocols.

In this retrospective study, records of cervical cancer screening stored by Shenzhen Baoan Shiyan People’s Hospital, Shenzhen were collected to analyse trends on local cervical cancer occurrence and the related HPV infections. Regional HPV type distribution and its annual changes were summarised. Cancer developing risks on different HPV type infections and coinfections were investigated with cytologic and histologic results. Finally, advices for region specific cervical cancer prevention were conclude and discussed.

## Methodology

### Patient enrolment criteria and screening protocol

Cervical cancer screening data from 2017.01 to 2023.05 were collected from Shenzhen Baoan Shiyan People’s Hospital, Shenzhen and the related community health centres. Patients included were restricted to those of age 18 to 75 with a history of sexual activity and willingly received screening related tests. In this retrospective study, all patients remained anonymous in data analysis so that need for patients’ consent was waived. The study process was in accordance with the Declaration of Helsinki, approved by the Ethics Committee of Shenzhen Baoan Shiyan People’s Hospital. Patients were first tested by HPV genotyping or ThinPrep cytologic tests (TCTs) or both. Those with high-risk HPV type infections or abnormal cytologic results would be recommended further testing with colposcopies where biopsy could be performed when visual lesions were found.

### Cervical sample collection

Cervical cells were collected by the patient or a certified clinician. After wiping cervix, the cervical brush was immediately washed with ThinPrep PreservCyt solutions. The cervical cells preserved by the solution would be used for HPV genotyping and TCT within 48 h.

### HPV DNA genotyping

HPV genotypes were detected with PCR-reverse dot hybridization. Three specialised HPV genotyping panels (see supplement for details) from Yaneng Bioscience (Human Papillomavirus kit and Human Papillomavirus kit for 23 types) and Hybribio (21 HPV GenoArray Diagnostic kit) were used. Sample DNA was extracted, amplified and then hybridised in automatic hybridization platform (Yaneng Bioscience, Shenzhen) with panels mentioned according to manufacture constructions. These three panels used in different time for different groups of population cover different types of HPV, but there are 15 types of HPV tested by all three panels. Totally 24 HPV variant types were tested, but only the overlapped 15 main types, including 6, 11, 16, 18, 31, 33, 35, 39, 45, 51, 52, 56, 58, 59 and 68, were intensively analysed in this study. When investigating multiple type coinfection, coinfections of more than 3 types were considered as combinations of multiple coinfection pairs. The coinfection rate was the proportion of coinfection cases within total infections of certain HPV type. Meanwhile, the coinfection coefficient was obtained by dividing coinfection case number by the product of total infection numbers of the two types within the coinfection pair.

### ThinPrep cytologic test

Cervical slides were prepared with a ThinPrep 2000 PROCESSOR. These slides would then be analysed and classified by experienced pathologists according to Bethesda 2001 criteria. Four classes interpretations: negative for intraepithelial lesion or malignancy (NILM); atypical squamous cells of uncertain significance (ASC-US); low-grade squamous intraepithelial lesion (LSIL) and high-grade squamous intraepithelial lesion (HSIL) were included in this study.

### Colposcopy guided biopsy

Colposcopy would be used to examine patients with high-risk HPV infection or abnormal cytological results. The operations were performed with SLC-3000 system where tissue sample would be taken when visual abnormalities were found. Samples were then examined by pathologists and classified accordingly. The results include no lesion, cervical intra-epithelial neoplasia (CIN1 ~ 3) and cervical cancer. In this study, lesions worse than CIN2 were considered high-grade CIN (hCIN) while other lesions were considered low-grade CIN (lCIN).

### Statistical analysis

Statistical analyses were performed with R 3.6 and python 3.6. Results of different tests reported within 90 days were considered assessments toward the same health state. Confident intervals were calculated with Wilson score. Significant level applied was 0.05 if not specified.

## Results

### HPV infection and cervical lesion overview

Records collected from multiple institutes could contain duplications and missing information. After careful data cleaning, 63,906 patients were included by this study. Results of 70,056 HPV genotyping tests, 74,008 TCTs, 10,238 colposcopies and 8716 biopsies were included and analysed (Table 1). Of all the HPV tests, 19,543 were reported positive and the most frequently detected HPV types amid were 52, 16, 6, 51 and 58. HPV infections were mostly detected within age strata 30–39 (supplement table). Meanwhile, within 74,008 TCTs preformed, 10,923 reported abnormal results. 10,238 colposcopies were conducted generating 8,716 biopsy samples, of which 814 were diagnosed and confirmed as high-grade CIN and the majority of samples showed low-grade CIN. Some participants involved were tested for routine body check, but others received tests for having gynecologic disease related symptoms. Thus, the positive rate observed in this research could be higher than the actual rate of all related women in the area. Number of colposcopies preformed was less than number of high-risk HPV infection and abnormal cytologic results, which was probably cause by patients rejecting the tests when the urge was not imperious.

**Table 1:** Screen data overview

Patient involved	63,906
Year of birth:	
Before 1960	664
1960–1969	4104
1970–1979	15,044
1980–1989	27,849
1990–2000	16,680
After 2000	807
HPV tests	70,056
Positive	19,543
Negative	50,513
Patients tested	47,435
TCT tests	74,008
NILM	63,085
ASC-US	8844
LSIL	1702
HSIL(ASC-H)	377
Patients tested	50,594
Colposcopy	10,238
Neg	2370
lCIN	7518
hCIN	350
Patients examined	8650
Biopsy	8716
Neg	1702
lCIN	6200
hCIN	814
Patients examined	6689

Abbreviations: NILM, negative for intraepithelial lesion or malignancy; ASC-US, atypical squamous cells of undetermined significance; LSIL, low-grade squamous intraepithelial lesion; HSIL, high-grade squamous intraepithelial lesions; lCIN, low-grade cervical intraepithelial neoplasia; hCIN, high-grade cervical intraepithelial neoplasia; Neg, negative.

### HPV distribution trend by year

**Fig. 1:**
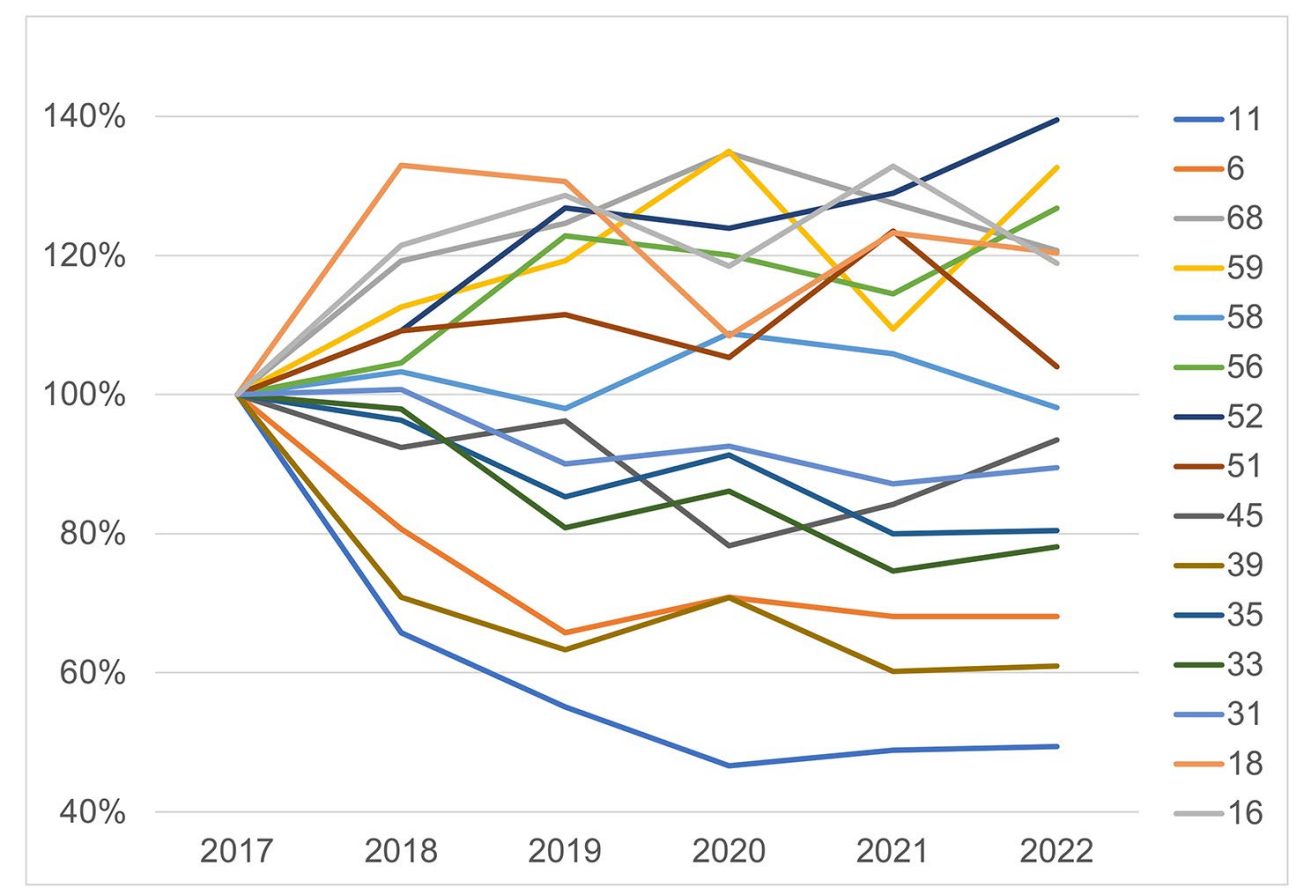
Annual changes of type proportion in HPV infections

HPV genotyping results were categorised by year and the data of different HPV type infections in the first year (2017) were used as baseline to calculate changes in proportion in the following years. As illustrated in Fig. 1, among those detected infections, the proportion of HPV 52, 59, 68, 56, 51, 16 and 18 rose above the first year throughout the entire period of this study, while that of HPV 58, 31 and 45 mainly remained the same with some fluctuations and the rest decreased. Most significant changes occurred in 2020 and 2021, which could be the result of radical changes of traveling frequency due to strict quarantines for SARS-CoV-2. In terms of the main HPV types of the region, the annual HPV type distribution trends indicated that the top types of HPV 52, 16 and 51 would remain in the future. It is worth noticing that HPV 16 is also the most carcinogenic type of HPV bringing significant challenges to cervical cancer prevention.

### Lesions detected by TCT and HPV infection

TCT and HPV genotyping test could both be applied in the beginning of screening. However, TCTs were applied more widely in the region studied, which was reflected on the number of tests conducted. TCT results and HPV genotyping results of those with confirmed lesions were compared in Table 2. Amid the 4771 low-grade CIN samples, 1406 of them were tested normal by TCT and 1486 of them were tested negative for HPV. Meanwhile, of the 763 high-grade CIN samples, 232 were tested normal by TCT but only 30 were tested negative for HPV. This indicated that compared to TCT, HPV genotyping might be slightly less sensitive to lower grade lesions but far more sensitive to high-grade lesions including cancers. In more general terms, TCT and HPV genotyping are similarly sensitive toward cervical lesions but HPV genotyping is notably more sensitive toward cervical cancer according to the data in this study. Another fact worth noticing is that, the proportion of HPV 16 infection was significantly higher in high-grade CIN samples, which is in accord with the consensus of it being the most carcinogenic HPV. However, no strong relation was found between certain HPV type infections and the inconsistence of cytologic and histologic results.

**Table 2:** TCT and HPV genotyping results of lesion cases

Diagnosis (no. of case)	TCT result	TCT number	HPV+	3 most observed (no. of case)
lCIN (4771)	NILM	1406	1227	52(334), 16(212), 58(134)
	ASC-US	2719	1531	52(459), 58(204), 16(198)
	LSIL	570	463	52(111), 51(85), 58(64)
	HSIL	76	64	52(23), 58(12), 51(10)
hCIN (763)	NILM	232	223	16(83), 52(57), 58(36)
	ASC-US	313	296	16(81), 52(78), 51(49)
	LSIL	103	102	52(29), 16(22), 58(19)
	HSIL	115	112	16(51), 52(31), 58(16)

Abbreviations: HPV+, positive for HPV test; NILM, negative for intraepithelial lesion or malignancy; ASC-US, atypical squamous cells of undetermined significance; LSIL, low-grade squamous intraepithelial lesion; HSIL, high-grade squamous intraepithelial lesions; lCIN, low-grade cervical intraepithelial neoplasia; hCIN, high-grade cervical intraepithelial neoplasia.

### HPV coinfection analysis

**Fig. 2:**
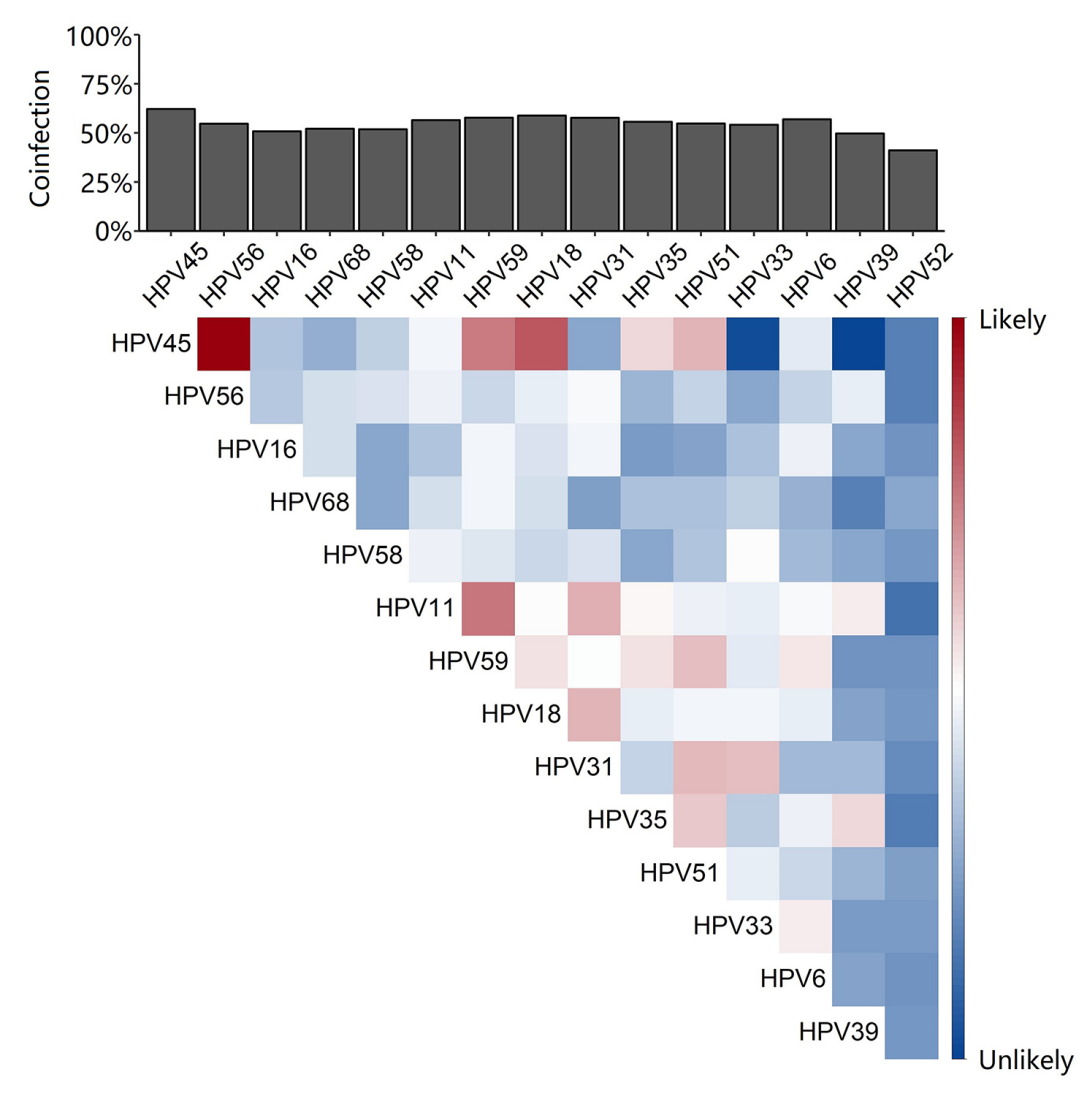
Coinfection coefficient heatmap. The likeliness of coinfection between HPV types is represented by red and blue. The overall coinfection rates observed in this study is presented on the top

To understand coinfection of different HPV types, cases infected by more than one HPV type were analysed and coinfection coefficients and coinfection rates were calculated. The coinfection coefficient was designed to normalise effects by type distibution and therefore presenting the relative effects of interactions between the 2 HPV types on infection. On the other hand, the coinfection rates of the main HPV types reflect the overall effect of coinfection in the region. Coinfection coefficients of the 15 main HPV types were illustrated in Fig. 2 with the corresponding coinfection rates showing that HPV types of high risk or most common, such as HPV 16, 52 and 58, are unlikely to coinfect with other types of HPV. In comparison, low risk HPV types, such as HPV 42, 43 and 66, are more likely to coinfect with others. This indicates that coinfection is unlikely to increase risk of being infected by carcinogenic HPV.

### Risk of HPV coinfection

We further compared coinfection to single HPV type infection on developing hCIN. Case number of single type infection and that of hCIN were listed on Table 3 with single infection hCIN (si-hCIN) rates which were obtained by dividing the hCIN number by the single infection number. It showed that HPV 16 has the highest si-hCIN rate followed by HPV 33, 58 and 18. Interestingly, HPV 18 was considered as carcinogenic as HPV 16 in most screening protocol but this study suggested HPV 33 and 58 are with similar, if not higher, risk as HPV 18. On the other hand, HPV 6 and 11 were lowest in si-hCIN rate although they are among the few HPV types direct covered by certain HPV vaccines.

**Table 3:** HPV infection of high-grade CIN case

type	single-infected	si-hCIN	si-hCIN-rate
6	653	3	0.0046
11	377	4	0.0106
16	884	204	0.2308
18	323	35	0.1084
31	152	14	0.0921
33	271	38	0.1402
35	119	8	0.0672
39	343	12	0.0350
45	77	4	0.0519
51	657	47	0.0715
52	1928	132	0.0685
56	318	16	0.0503
58	665	73	0.1098
59	321	12	0.0374
68	506	20	0.0395
Neg	49,881	140	0.0028

Abbreviations: hCIN, high-grade cervical intraepithelial neoplasia; si-hCIN: single infection hCIN.

The hCIN rates of most frequently found coinfection pairs were calculated and compared to the estimated coinfection high-grade CIN rates on Table 4. The estimated coinfection hCIN rate was the union of the related si-hCIN rates. Only coinfection pairs with more than 30 cases were displayed on the table. The fact of actual rates being lower than the estimated rates indicates that, according to the data of listed pairs in this study, coinfections reduced the risk of developing high-grade CIN. Although there were pairs with actual rate higher than estimated rate, such as 18–52 and 16–68 (data not show), these could be errors caused by small size sampling.

**Table 4:** High-grade CIN case rate of double type HPV infection

infectpair	infected	hCIN	hCIN-rate	est-rate
16–52	94	19	0.2021	0.2834
52–58	76	10	0.1316	0.1707
51–52	63	8	0.1270	0.1351
52–68	60	5	0.0833	0.1053
52 − 6	58	5	0.0862	0.0727
16 − 6	55	5	0.0909	0.2343
52–53	54	4	0.0741	0.0787
52–81	49	2	0.0408	0.0730
51 − 6	34	2	0.0588	0.0758

Abbreviations: hCIN, high-grade cervical intraepithelial neoplasia; est-rate, estimated rate.

## Discussion

### HPV distribution similar to most area in China

The most prevalent HPV types across China were reported to be 16, 18, 33, 52 and 58 in various orders [10]. However, the most detected HPV types in Baoan, Shenzhen were slightly different, where type 52, 58 and 16 were still the most common but infections of type 18 and 33 were less than that of type 6 and 51. Similar type profiles were reported in Zhejiang [11], Jiangsu [12] and Beijing [13]. These provinces or cities are all closely connected with each other economically including Shenzhen which could lead to stable population circulations among these regions and therefore similar HPV type distribution. Meanwhile, most cervical cancer cases in Shenzhen were associated to the infections of HPV 16 and 52, which is significantly different from which reported in other countries [14] or worldwide scale [15] but is consistent with researches conducted in China [16]. These researches showed that the overall HPV type distribution could be highly region specific and would fundamentally affect the prevalence of cervical cancer.

Apart from the overall type distributions, the trends of annual changes in these distributions were also analysed. It appeared that the main high-risk HPV types were all gradually rising in proportion, indicating potential regional raise of cervical cancer risk in the upcoming years. Vaccination is one of the main approaches for reducing HPV infection and risk of developing cervical cancers but the vaccination coverage in China was low [17]. It was reported that the complete vaccination rate in China was around 3% in 2020 [17] and the rate of receiving at least one injection in Shenzhen was 18.7% in 2023 [18]. Of the 3 vaccines available in Shenzhen in 2023, only the nonavalent vaccine covers more commonly detected types (HPV 52/58) in the area and therefore could be the best for countering HPV infection in China [19]. However, considering the low availability of the nonavalent vaccines and the fact that most carcinogenic HPV 16 is covered by all three vaccines available, administration of the other 2 vaccines should also be promoted for regional cervical cancer prevention. Significant efforts has been invested into vaccine development to increase availability [20] but it is largely behind the increasing requirement of covering the main types in China [9–11].

### Progressions on cervical screening protocol in China

By including colposcopy results of patients infected by high-risk HPV, this study was able to investigate the sensitivity of TCT and HPV genotyping test toward cervical lesions. It showed that TCT was slightly more sensitive for detecting general cervical lesions than HPV test, which was consistent with other researches [21, 22]. However, as for hCIN case detections, HPV tests were significantly more sensitive, suggesting HPV infection being a better cervical cancer indicator. Similar comparative results were observed in other researches [14, 23] and some researchers further suggested to implement HPV genotyping without TCTs as primary test in cervical cancer screening [24].

The cervical cancer screening guideline in China established by the related Chinese expert panel adopted TCT and test for HPV 16/18 as the primary test where only patients with abnormal TCT results or HPV 16/18 infection would be further tested with colposcopy [25]. The updated 2023 guideline extended tests on persistent infections of other high-risk HPV types in the first-line screening where colposcopy would also be advised to patients with non-16/18 high-risk HPV infections over 6 months [26]. Institutes included by this study did not strictly follow the guide and expanded colposcopy test for all high-risk HPV infection. Although excessive colposcopy is not recommended, the results enable comparison of carcinogenicity between HPV types. This study demonstrated that a higher proportion of cervical cancers were associated with HPV 52/58 compared to HPV 18 and the hCIN rate of HPV 33 infection was significantly higher than that of HPV 18, suggesting the need for expanding HPV genotyping in cervical cancer screening. Similar results were observed by researches conducted in China [27, 28] and the researchers advised HPV genotyping should be expanded to include certain high-risk HPVs separately [27] or as a group [28]. Meanwhile, Cuzick and Wheeler emphasised the predictive value of HPV 31/33 while pointed out the comparatively lower risk of some high-risk HPV types based on the result of a nation-wide survey in America [29]. All these researches indicated the need for a more detailed triage strategy based on HPV genotyping for cervical cancer screening. Expansion on persistent HPV infection on screening guideline of China also expressed a motive on such direction.

### HPV coinfection and risk

HPV coinfection and the consequential risk were also investigated in this research and it showed that high-risk HPV types are less likely to coinfect compared to the low-risk types and the cancer risks are likely to decrease in coinfections. Many researches [30, 31] reported the most common coinfection types and some [32–34] even claimed these types were more likely to coinfect based on these observation, discounting the respective infecting rates of the related types. These infection rates of different HPV types was considered in this study and the results indicated that high frequencies of coinfection of certain types were likely resulted from high respective infection rates of the related types and different conclusions could be drawn when the frequencies were normalised by these infection rates.

As for the effect of coinfections on cancer risk, many researches associated coinfections with cytological results and claimed that coinfections contributed to a higher cytologic abnormality rate [31, 35, 36]. Meanwhile, other teams correlating coinfections with histological results concluded that coinfections would not increase risks of developing cancers [37–39]. The results obtained on this research support the latter, but the data involved was not sufficient to draw a decisive conclusion.

### Research limitations

A major limitation of this study was the normality and integrity of records. Patients were taking tests spontaneously but not by proper schedule so that relations between different tests were not entirely certain. Besides, patients might receive tests or treatments or vaccines from institutes not included by this study and the related records could not be acquired by this study. Although the issues described should only apply to a small proportion of data involved, the effects to final results remain unknown. Therefore, although hypotheses generated in this study were supported by real-world data, verifications with carefully designed experiments should be conducted in the future.

## Conclusion

The cervical cancer screening records from Baoan, Shenzhen showed that HPV 52, 58, 16 were the most detected types while HPV 16 was the most carcinogenetic. The data also indicated that HPV genotyping is more sensitive toward high-grade CIN compared to TCT and HPV coinfections are not likely to increase risk for cervical cancers.

### Electronic supplementary material

Below is the link to the electronic supplementary material.

Supplementary Material 1

## Data Availability

All data generated or analyzed during this study are included in this published article and its supplementary information files.

## Abbreviations

HPV: Human papillomavirus
HPV+: HPV-positive
CIN: cervical intra-epithelial neoplasia
TCT: ThinPrep cytologic test
NILM: negative for intraepithelial lesion or malignancy
ASC-US: atypical squamous cells of uncertain significance
ASC-H: typical squamous cells, not excluding high-grade squamous intraepithelial lesion
LSIL: low-grade squamous intraepithelial lesion
HSIL: high-grade squamous intraepithelial lesion
hCIN: high-grade CIN
lCIN: low-grade CIN
si-hCIN: single infection hCIN

## References

[1] Bhatla N, Aoki D, Sharma DN, Sankaranarayanan R. Cancer of the cervix uteri. Int J Gynecol Obstet. 2018;143:22–36. 10.1002/ijgo.12611. 10.1002/ijgo.12611 30306584

[2] Sung H, Ferlay J, Siegel RL, et al. Global Cancer statistics 2020: GLOBOCAN estimates of incidence and Mortality Worldwide for 36 cancers in 185 countries. CA Cancer J Clin. 2021;71(3):209–49. 10.3322/caac.21660. 33538338 10.3322/caac.21660

[3] Cao W, Chen H, Da, Yu YW, Li N, Chen WQ. Changing profiles of cancer burden worldwide and in China: a secondary analysis of the global cancer statistics 2020. Chin Med J (Engl). 2021;134(7):783–91. 10.1097/CM9.0000000000001474. 33734139 10.1097/CM9.0000000000001474 PMC8104205

[4] WHO. Evidence-Based Consensus Recommendations for Colposcopy Practice for Cervical Cancer Prevention in the United States. second edi. https://www.who.int/publications/i/item/9789240030824. 10.1097/LGT.0000000000000322 28953109

[5] Kessler TA. Cervical Cancer: Prevention and early detection. Semin Oncol Nurs. 2017;33(2):172–83. 10.1016/j.soncn.2017.02.005. 28343836 10.1016/j.soncn.2017.02.005

[6] Kogure G, Onuki M, Hirose Y, et al. Whole-genome analysis of human papillomavirus 67 isolated from Japanese women with cervical lesions. Virol J. 2022;19(1):1–8. 10.1186/s12985-022-01894-z. 36207729 10.1186/s12985-022-01894-z PMC9547447

[7] Athanasiou A, Bowden S, Paraskevaidi M, et al. HPV vaccination and cancer prevention. Best Pract Res Clin Obstet Gynaecol. 2020;65:109–24. 10.1016/j.bpobgyn.2020.02.009. 32284298 10.1016/j.bpobgyn.2020.02.009

[8] Bruni L, Serrano B, Roura E, et al. Cervical cancer screening programmes and age-specific coverage estimates for 202 countries and territories worldwide: a review and synthetic analysis. Lancet Glob Heal. 2022;10(8):e1115–27. 10.1016/S2214-109X(22)00241-8. 10.1016/S2214-109X(22)00241-8 PMC9296658 35839811

[9] Wei L, Xie X, Liu J, et al. Elimination of Cervical Cancer: challenges promoting the HPV Vaccine in China. Indian J Gynecol Oncol. 2021;19(3):8–11. 10.1007/s40944-021-00536-6. 10.1007/s40944-021-00536-6 PMC8236217 34222614

[10] Li K, Li Q, Song L, Wang D, Yin R. The distribution and prevalence of human papillomavirus in women in mainland China. Cancer. 2019;125(7):1030–7. 10.1002/cncr.32003. 30748006 10.1002/cncr.32003

[11] Yan X, Shen L, Xiao Y, Wang Q, Li F, Qian Y. Prevalence, characteristics, and distribution of HPV genotypes in women from Zhejiang Province, 2016–2020. Virol J. 2021;18(1):1–12. 10.1186/s12985-021-01676-z. 34670576 10.1186/s12985-021-01676-z PMC8527678

[12] Wang T, Luan L, Deng J, et al. Prevalence and human papillomavirus (HPV) genotype distribution in Suzhou, China. Hum Vaccines Immunother. 2023;19(2). 10.1080/21645515.2023.2241309. 10.1080/21645515.2023.2241309 PMC10392751 37519009

[13] Zhu X, Wang Y, Lv Z, Su J. Prevalence and genotype distribution of high-risk HPV infection among women in Beijing, China. J Med Virol. 2021;93(8):5103–9. 10.1002/jmv.27013. 33847386 10.1002/jmv.27013

[14] Monsonego J, Cox JT, Behrens C, Sandri M, Franco EL, Yap PS, et al. Prevalence of high-risk human papilloma virus genotypes and associated risk of cervical precancerous lesions in a large U.S. screening population: data from the ATHENA trial. Gynecol Oncol. 2015;137(1):47–54. 10.1016/j.ygyno.2015.01.551. 25667973 10.1016/j.ygyno.2015.01.551

[15] De Sanjose S, Quint WGV, Alemany L, Geraets DT, Klaustermeier JE, Lloveras B, et al. Human papillomavirus genotype attribution in invasive cervical cancer: a retrospective cross-sectional worldwide study. Lancet Oncol. 2010;11(11):1048–56. 10.1016/S1470-2045(10)70230-8. 20952254 10.1016/S1470-2045(10)70230-8

[16] Tao X, Austin RM, Yu T, et al. Risk stratification for cervical neoplasia using extended high-risk HPV genotyping in women with ASC-US cytology: a large retrospective study from China. Cancer Cytopathol. 2022;130(4):248–58. 10.1002/cncy.22536. 34874615 10.1002/cncy.22536

[17] Wang L, Zhong Y, Di J. Current experience in HPV Vaccination in China. Indian J Gynecol Oncol. 2021;19(3). 10.1007/s40944-021-00535-7.

[18] Lin Z, Liang X, Su L, Peng W, Chen H, Fang Y, et al. Coverage with the first dose of human papillomavirus vaccination among females aged 9–50 years in Shenzhen, China: a Surveillance based on Administrative Health Records in 2023. Vaccines. 2024;12(1):1–10. 10.3390/vaccines12010075. 10.3390/vaccines12010075 PMC10818281 38250888

[19] Joura EA, Giuliano AR, Iversen OE, et al. A 9-Valent HPV vaccine against infection and Intraepithelial Neoplasia in Women. N Engl J Med. 2015;372(8):711–23. 10.1056/nejmoa1405044. 25693011 10.1056/nejmoa1405044

[20] Wang D, Liu X, Wei M, et al. Rational design of a multi-valent human papillomavirus vaccine by capsomere-hybrid co-assembly of virus-like particles. Nat Commun. 2020;11(1):1–15. 10.1038/s41467-020-16639-1. 32503989 10.1038/s41467-020-16639-1 PMC7275066

[21] Zhang YY, Xu XQ, Zhang D, Wu J, Zhang HX. Triage human papillomavirus testing for cytology-based cervical screening in women of different ages in primary hospitals: a retrospective clinical study. Med (United States). 2020;99(38):E22320. 10.1097/MD.0000000000022320. 10.1097/MD.0000000000022320 PMC7505382 32957398

[22] Husaiyin S, Jiao Z, Yimamu K, Maisaidi R, Han L, Niyazi M. ThinPrep cytology combined with HPV detection in the diagnosis of cervical lesions in 1622 patients. PLoS ONE. 2021;16(12 December):1–11. 10.1371/journal.pone.0260915. 10.1371/journal.pone.0260915 PMC8638999 34855928

[23] Wang L, Song Q, Liu Y, Ou Q. ThinPrep cytologic test combined with HPV typing to evaluate the degree of cervical diseases and the relationship between HPV typing and the pathological results of patients with atypical squamous cells of undetermined significance: a diagnostic test. Transl Cancer Res. 2022;11(9):3277–86. 10.21037/tcr-22-2026. 36237241 10.21037/tcr-22-2026 PMC9552063

[24] Wright TC, Stoler MH, Behrens CM, Sharma A, Zhang G, Wright TL. Primary cervical cancer screening with human papillomavirus: end of study results from the ATHENA study using HPV as the first-line screening test. Gynecol Oncol. 2015;136(2):189–97. 10.1016/j.ygyno.2014.11.076. 25579108 10.1016/j.ygyno.2014.11.076

[25] CSCCP. Expert consensus on cervical cancer screening and abnormal management in China (Part I). Chin J Clin Obstet Gynecol. 2017;18(02):190–2. 10.13390/j.issn.1672-1861.2017.02.032. 10.13390/j.issn.1672-1861.2017.02.032

[26] Branch of Cancer Prevention and Control CPMA, Chinese Obstetrics and Gynecology Association Colposcopy and Cervical Neoplasia Committee, Chinese Society of Colposcopy and Cervical Pathology of China Health Birth Science Association. Beijing Medical Doctor (Technician) Society of Laboratory Medicine. [Chinese expert consensus on the use of human papillomavirus nucleic acid testing for cervical cancer screening (2022)]. Zhonghua Yi Xue Za Zhi. 2023;103(12):1184–95. 10.3760/cma.j.cn112137-20230117-00096. 37062594 10.3760/cma.j.cn112137-20230117-00096

[27] Dong L, Hu SY, Zhang Q, Feng RM, Zhang L, Zhao XL, et al. Risk prediction of cervical cancer and precancers by type-specific human papillomavirus: evidence from a population-based cohort study in China. Cancer Prev Res. 2017;10(12):745–51. 10.1158/1940-6207.CAPR-17-0088. 10.1158/1940-6207.CAPR-17-0088 28916509

[28] Song F, Du H, Xiao A, Wang C, Huang X, Liu Z, et al. Type-specific distribution of cervical hrHPV infection and the association with cytological and histological results in a large population-based cervical cancer screening program: baseline and 3-year longitudinal data. J Cancer. 2020;11(20):6157–67. 10.7150/jca.48357. 32922555 10.7150/jca.48357 PMC7477419

[29] Cuzick J, Wheeler C. Need for expanded HPV genotyping for cervical screening. Papillomavirus Res. 2016;2:112–5. 10.1016/j.pvr.2016.05.004. 29074170 10.1016/j.pvr.2016.05.004 PMC5886893

[30] Kim M, Park NJY, Jeong JY, Park JY. Multiple human papilloma virus (hpv) infections are associated with hsil and persistent hpv infection status in Korean patients. Viruses. 2021;13(7):4–14. 10.3390/v13071342. 10.3390/v13071342 PMC8310096 34372548

[31] Kim J, Kim M, Park JY. Evaluation of the characteristics of multiple human papillomavirus (HPV) infections identified using the BD Onclarity HPV assay and comparison with those of single HPV infection. J Pathol Transl Med. 2022;56(5):289–93. 10.4132/JPTM.2022.08.02. 36128865 10.4132/JPTM.2022.08.02 PMC9510038

[32] Chaturvedi AK, Katki HA, Hildesheim A, et al. Human papillomavirus infection with multiple types: pattern of coinfection and risk of cervical disease. J Infect Dis. 2011;203(7):910–20. 10.1093/infdis/jiq139. 21402543 10.1093/infdis/jiq139 PMC3068034

[33] Gallegos-Bolaños J, Rivera-Domínguez JA, Presno-Bernal JM, Cervantes-Villagrana RD. High prevalence of co-infection between human papillomavirus (HPV) 51 and 52 in Mexican population. BMC Cancer. 2017;17(1):1–8. 10.1186/s12885-017-3519-7. 28789619 10.1186/s12885-017-3519-7 PMC5549346

[34] Liao G, Jiang X, She B, et al. Multi-infection patterns and co-infection preference of 27 human papillomavirus types among 137,943 gynecological outpatients across China. Front Oncol. 2020;10(April):1–9. 10.3389/fonc.2020.00449. 32318343 10.3389/fonc.2020.00449 PMC7154087

[35] Kim SY, Hwang KA, Ann JH, Kim JH, Nam JH. Next-generation sequencing for typing human papillomaviruses and predicting multi-infections and their clinical symptoms. Microbiol Immunol. 2021;65(7):273–8. 10.1111/1348-0421.12927. 34133044 10.1111/1348-0421.12927

[36] Carrillo-García A, Ponce-de-León-Rosales S, Cantú-de-León D, et al. Impact of human papillomavirus coinfections on the risk of high-grade squamous intraepithelial lesion and cervical cancer. Gynecol Oncol. 2014;134(3):534–9. 10.1016/j.ygyno.2014.06.018. 24979052 10.1016/j.ygyno.2014.06.018

[37] Song F, Yan P, Huang X, et al. Roles of extended human papillomavirus genotyping and multiple infections in early detection of cervical precancer and cancer and HPV vaccination. BMC Cancer. 2022;22(1):1–13. 10.1186/s12885-021-09126-3. 34991494 10.1186/s12885-021-09126-3 PMC8734293

[38] Salazar KL, Zhou HS, Xu J, et al. Multiple human papilloma virus infections and their impact on the development of high-risk cervical lesions. Acta Cytol. 2015;59(5):391–8. 10.1159/000442512. 26674365 10.1159/000442512

[39] Senapati R, Nayak B, Kar SK, Dwibedi B. HPV genotypes co-infections associated with cervical carcinoma: special focus on phylogenetically related and non-vaccine targeted genotypes. PLoS ONE. 2017;12(11):1–10. 10.1371/journal.pone.0187844. 10.1371/journal.pone.0187844 PMC5697876 29161285

